# Timing of physical therapy for individuals with patellofemoral pain and the influence on healthcare use, costs and recurrence rates: an observational study

**DOI:** 10.1186/s12913-021-06768-8

**Published:** 2021-07-29

**Authors:** Jodi L. Young, Suzanne J. Snodgrass, Joshua A. Cleland, Daniel I. Rhon

**Affiliations:** 1 Doctor of Science in Physical Therapy, Bellin College, 3201 Eaton Rd, Green Bay, WI 54311 USA; 2 Discipline of Physiotherapy, School of Health Sciences, The University of Newcastle, University Dr, Callaghan, NSW 2308 Australia; 3 Doctor of Physical Therapy Program, Department of Public Health and Community Medicine, Tufts University School of Medicine, 419 Boston Ave, Medford, MA 02155 USA; 4 Center for the Intrepid, Brooke Army Medical Center, JBSA Fort Sam Houston, 3551 Roger Brooke Dr, San Antonio, TX 78234 USA; 5 Department of Rehabilitation Medicine, Uniformed Services University of Health Sciences, 4301 Jones Bridge Rd, Bethesda, MD 20814 USA

**Keywords:** Exercise therapy, Physical therapy, Healthcare utilization

## Abstract

**Background:**

Early physical therapy has been shown to decrease downstream healthcare use, costs and recurrence rates in some musculoskeletal conditions, but it has not been investigated in individuals with patellofemoral pain. The purpose was to evaluate how the use and timing of physical therapy influenced downstream healthcare use, costs, and recurrence rates.

**Methods:**

Seventy-four thousand four hundred eight individuals aged 18 to 50 diagnosed with patellofemoral pain between 2010 and 2011 in the Military Health System were categorized based on use and timing of physical therapy (first, early, or delayed). Healthcare use, costs, and recurrence rates were compared between the groups using descriptive statistics and a binary logit regression.

**Results:**

The odds for receiving downstream healthcare use (i.e. imaging, prescription medications, and injections) were lowest in those who saw a physical therapist as the initial contact provider (physical therapy first), and highest in those who had delayed physical therapy (31–90 days after patellofemoral pain diagnosis). Knee-related costs for those receiving physical therapy were lowest in the physical therapy first group ($1,136, 95% CI $1,056, $1,217) and highest in the delayed physical therapy group ($2,283, 95% CI $2,192, $2,374). Recurrence rates were lowest in the physical therapy first group (AOR = 0.55, 95% CI 0.37, 0.79) and highest in the delayed physical therapy group (AOR = 1.78, 95% CI 1.36, 2.33).

**Conclusions:**

For individuals with patellofemoral pain using physical therapy, timing is likely to influence outcomes. Healthcare use and costs and the odds of having a recurrence of knee pain were lower for patients who had physical therapy first or early compared to having delayed physical therapy.

## Background

Patellofemoral pain (PFP) has an annual prevalence of approximately 23% in the general population [1], affecting both adolescents and adults [2]. Over a lifetime, 13% of adults will see a general practitioner for knee pain, and 6.8% will be referred to a secondary provider [3]. A formal diagnosis of PFP is often slow due to the progressive onset of symptoms [4]. The diagnosis is based on a cluster of signs and symptoms [4]; consequently, there are significant healthcare use and costs associated with PFP [2]. Physical therapy is one of the core recommendations in clinical practice guidelines [4, 5].

Various delivery models for physical therapy exist, including seeing a physical therapist at the initial contact with the health system (i.e. direct access), as an early referral as part of an initial intervention strategy, or as a delayed referral, often after other options may have failed [6]. Evidence suggests that timing is important, and appropriate physical therapy interventions delivered earlier compared to later in the management pathway can diminish the likelihood of unnecessary imaging and pharmacological management [7]. There is considerable evidence for decreased long-term healthcare use and costs when physical therapy is received early [7–9], but these data are primarily from individuals with spinal pain and it is unknown if the same outcomes would be expected in patients with PFP.

Similar to low back pain, recurrence of symptoms and chronic pain are common in individuals with PFP [10, 11]. Unlike low back pain, where evidence supports a reduction in recurrence with early and appropriate physical therapy [9], it is unknown if the use and timing of physical therapy influences recurrence in patients with PFP. A recent study found that prior opioid use led to a greater number of recurrences in patients with PFP [12], but did not address the use or timing of physical therapy.

The purpose of the study was to assess how the use and timing of physical therapy influenced downstream healthcare use and costs in individuals seeking care for PFP. A secondary objective was to determine if the timing of physical therapy was associated with recurrences of knee pain. The hypothesis was that delayed physical therapy would increase long-term healthcare use and costs and the odds of having a recurrence of knee pain.

## Methods

### Study design

This was an observational cohort study using data from patients in the Military Health System (MHS) receiving care for PFP in civilian and military clinics around the world. The Institutional Review Board of the Army Regional Health Command Central in San Antonio, TX, USA granted ethics and regulatory approval, and all methods were carried out in accordance with relevant guidelines and regulations. The need for patient consent was waived by the Institutional Review Board of the Army Regional Health Command Central because data were collected retrospectively and provided de-identified to the research team. To guide reporting of this study, the REporting of studies Conducted using Observational Routinely-collected health Data (RECORD) statement [13], an extension of the Strengthening the Reporting of Observational Studies in Epidemiology (STROBE) statement [14] was used.

### Data source

Data were acquired from the monthly updated MHS Data Repository (MDR), a centralized data repository capturing Defense Health Agency (DHA) corporate healthcare data worldwide [15]. This includes all person-level data for all medical visits, laboratory procedures, and all medication prescriptions [15]. Data were provided de-identified and summed for each care variable for each individual.

### Participants

Individuals diagnosed with PFP in the MHS, using the International Classification of Disease, 9th revision (ICD-9) code 717.7 chondromalacia patellae, between January 1, 2010 and December 31, 2011 were included. This code was the most appropriate because there are no other non-traumatic patellar-related ICD-9 codes, it is the only label available to clinicians for use with all subtypes of PFP. Furthermore, subgroup diagnostic labels (i.e. anterior knee pain, jumper’s knee, patellofemoral pain syndrome, and chondromalacia patella often have poor validity and reliability and are unlikely to change treatment or prognosis, which is why recent guidelines recommend the use of the term PFP for all of these conditions collectively [16]. Individuals needed to be eligible for care at least 1 year prior and 2 years after diagnosis. Details of the development of the cohort have been published [17].

### Study variables

#### Descriptive variables

Age, sex, military service branch, and socioeconomic status (junior enlisted, senior enlisted, junior officer, senior officer) were presented according to those who received physical therapy first (on the same day as PFP diagnosis), early (from one to 30 days after initial PFP diagnosis), delayed (from 31 to 90 days after initial PFP diagnosis), or did not receive physical therapy.

#### Independent variables

Physical therapy use was defined as receiving an initial evaluation for the knee by a physical therapist within the 90 day period following the initial PFP diagnosis date. If an individual received physical therapy on the date of the diagnosis (i.e., when diagnosed by the physical therapist as first-contact practitioner, and considered day zero), they were categorized as receiving physical therapy first. Individuals who received physical therapy any time from day one to 30 after initial diagnosis were classified as receiving early physical therapy, and any time from 31 to 90 days after initial diagnosis were classified as receiving delayed physical therapy. Individuals who received no physical therapy following diagnosis were also included.

#### Outcome variables

Healthcare use in the 2 year surveillance period after diagnosis was captured, including all knee-related procedures, knee radiographs, advanced imaging (magnetic resonance imaging (MRI), knee arthrograms, or computed tomography (CT)) scans), and pharmacological interventions. Pharmacological interventions included opioids, muscle relaxants, non-steroidal anti-inflammatory drugs (NSAIDs), and other analgesic prescriptions, as well as corticosteroid injections. The number of physical therapy visits for the knee, knee-related care visits, total healthcare costs, and total knee-related care costs were also examined. A recurrence was defined as any visit for knee-related care after a period of at least 60 days without any knee care. This definition is a standard used in this health system to characterize a new injury [18].

#### Comorbidities

Due to the potential impact on prognosis and healthcare use [15], care-seeking for the following comorbidities in the year prior was also captured: cardiometabolic syndrome, chronic pain, insomnia, depression, and substance abuse disorders.

### Statistical analysis

Descriptive statistics, including means, standard deviations or 95% confidence intervals (CIs), and frequencies, were calculated for baseline and outcome variables based on the three categories of physical therapy use (first, early or delayed) and no physical therapy. A binary logit regression was used to identify the odds of healthcare use variables within the 2 years after initial PFP diagnosis. Unadjusted odds ratios (OR) and adjusted (sex and comorbidities identified earlier) odds ratios (AOR) and 95% CIs are reported. A *p* value of less than 0.05 was chosen a priori for statistical significance for all analyses performed using SPSS Statistics Version 26.0 (IBM Corporation, Armonk, NY).

Early physical therapy definitions have varied anywhere from 48 to 72 h after diagnosis [19, 20] to 14 to 30 days [21, 22], and therefore a sensitivity analysis was conducted setting the threshold to 14 days for all groups. Approaches based on clinical practice guidelines and recommendations are expected to result in better outcomes, and since exercise is a core treatment recommendation, we conducted another sensitivity analysis adjusting for individuals who received only active (therapeutic exercise and/or therapeutic activity) versus passive interventions (e.g. modalities).

## Results

Of 74,408 individuals who met eligibility criteria, the mean age was 32.1 (7.7) years, 30.4% were female, 48.2% were in the Army, and 43.9% were junior enlisted. Baseline demographics are provided in Table 1.

**Table 1:** Demographics

	Full cohort (***n*** = 74,408)	Any physical therapy use (***n*** = 15,430)	Physical therapy first (***n*** = 3,591)	Early physical therapy (***n*** = 7,924)	Delayed physical therapy (***n*** = 3915)	No physical therapy (***n*** = 58,579)
***Age***, mean (SD)	32.1 (7.7)	31.9 (7.4)	30.4 (7.3)	32.2 (7.3)	32.6 (7.5)	32.1 (7.8)
18 to 30	35,095 (47.2)	7377 (47.8)	1992 (55.5)	3684 (46.5)	1701 (43.4)	27,512 (47.0)
31 to 40	26,943 (36.2)	5785 (37.5)	1209 (33.7)	3015 (38.0)	1561 (39.9)	21,019 (35.9)
41 to 50	12,370 (16.6)	2268 (14.7)	390 (10.9)	1225 (15.5)	653 (16.7)	10,048 (17.2)
***Female sex***	22,640 (30.4)	4540 (29.4)	702 (19.5)	2600 (32.8)	1238 (31.6)	18,019 (30.8)
***Branch of service***						
Army	35,885 (48.2)	6445 (41.8)	1795 (50.0)	2927 (36.9)	1723 (44.0)	29,273 (50.0)
Air Force	19,731 (26.5)	5358 (34.7)	710 (19.8)	3312 (41.8)	1336 (34.1)	14,219 (24.3)
Coast Guard	1689 (2.3)	316 (2.0)	58 (1.6)	182 (2.3)	76 (1.9)	1367 (2.3)
Marine Corps	5940 (8.0)	1080 (7.0)	437 (12.2)	416 (5.2)	227 (5.8)	4822 (8.2)
Navy	11,163 (15.0)	2231 (14.5)	591 (16.5)	1087 (13.7)	553 (14.1)	8898 (15.2)
***Socioeconomic status***						
Junior Enlisted	32,702 (43.9)	6793 (44.0)	1624 (45.2)	3481 (43.9)	1688 (43.1)	25,724 (43.9)
Senior Enlisted	26,854 (36.1)	5422 (35.1)	1072 (29.9)	2888 (36.4)	1462 (37.3)	21,297 (36.4)
Junior Officer	7847 (10.5)	1762 (11.4)	472 (13.1)	884 (11.2)	405 (10.3)	6035 (10.3)
Senior Officer	6587 (8.9)	1272 (8.2)	259 (7.2)	659 (8.3)	354 (9.0)	5293 (9.0)
Unknown	418 (0.6)	182 (1.2)	164 (4.6)	12 (0.2)	6 (0.2)	230 (0.4)

Reported as number (percentage) of patients unless otherwise specified

Of those in the cohort who saw a physical therapist (*n* = 15,430; 20.7% of total cohort), 3591 (23.3% of those receiving physical therapy) received physical therapy first, 7924 (51.3% of those receiving physical therapy) received early physical therapy, and 3915 (25.4% of those receiving physical therapy) received delayed physical therapy. There were 58,579 individuals (78.7% of the total cohort) who did not see a physical therapist. The mean number of physical therapy visits was 5.16 (SD 7.39) in the physical therapy first group, 6.36 (SD 8.01) in the early physical therapy group, and 6.67 (SD 8.08) in the delayed physical therapy group. The mean number of knee-related care visits in the 2 year follow up period was 6.55 (SD 9.26) for the physical therapy first group, 9.99 (SD 10.59) for the early physical therapy group, and 11.55 (SD 10.93) for the delayed physical therapy group. Patients who did not receive any physical therapy had a mean of 3.73 (SD 6.27) knee-related care visits.

The most common healthcare component used in the cohort was knee radiographs (49.8%), and the least common was analgesic prescription medication (i.e. non-opioid and non-NSAIDs) (2.7%). Of those receiving physical therapy, the physical therapy first group had the lowest total mean 2 year healthcare costs at $7610 (95% CI $7268, $7951), and delayed physical therapy had the highest total mean healthcare costs ($10,354 (95% CI $10,017, $10,691)). Mean 2 year total knee-related costs followed a similar pattern, with $1136 (95% CI $1056, $1217) for the physical therapy first group, and $2283 (95% CI $2192, $2374) for delayed physical therapy. Table 2 provides all use and cost outcomes in the 2 year time period after the initial PFP diagnosis.

**Table 2:** Healthcare use and cost outcomes in 2 year time period after diagnosis

	Full cohort (***n*** = 74,408)	Any physical therapy use (***n*** = 15,430)	Physical therapy first (***n*** = 3591)	Early physical therapy (***n*** = 7924)	Delayed physical therapy (***n*** = 3915)	No physical therapy (***n*** = 58,579)
***Number of individuals receiving imaging/pharmaceuticals, N (%)***						
Knee x-ray	37,058 (49.8)	9132 (59.2)	1045 (29.1)	5134 (64.8)	2953 (75.4)	27,669 (47.2)
Advanced imaging	16,166 (21.7)	4705 (30.5)	774 (21.6)	2255 (28.5)	1676 (42.8)	11,346 (19.4)
Opioids	4889 (6.6)	1502 (9.7)	193 (5.4)	770 (9.7)	539 (13.8)	3333 (5.7)
Muscle relaxants	2453 (3.3)	744 (4.8)	90 (2.5)	401 (5.1)	253 (6.5)	1677 (2.9)
NSAIDs prescription	6757 (9.1)	2121 (13.7)	253 (7.0)	1137 (14.3)	731 (18.7)	4562 (7.8)
Other analgesics (acetaminophen, etc.)	1984 (2.7)	629 (4.1)	80 (2.2)	328 (4.1)	221 (5.6)	1333 (2.3)
Corticosteroid injections	3233 (4.3)	995 (6.4)	128 (3.6)	512 (6.5)	355 (9.1)	2213 (3.8)
***Number of knee physical therapy visits, mean (SD)***	5.96 (7.92)	6.16 (7.91)	5.16 (7.39)	6.36 (8.01)	6.67 (8.08)	–
***Number of knee-related care visits, mean (SD)***	4.97 (7.75)	9.58 (10.54)	6.55 (9.26)	9.99 (10.59)	11.55 (10.93)	3.73 (6.27)
***Total healthcare costs, mean (95% CI)***	$8390 ($8310, $8470)	$9234 ($9069, $9398)	$7610 ($7268, $7951)	$9416 ($9193, $9639)	$10,354 ($10,017, $10,691)	$8160 ($8070, $8251)
***Total knee-related costs, mean (95% CI)***	$1103 ($1087, $1119)	$1821 ($1777, $1864)	$1136 ($1056, $1217)	$1902 ($1843, $1962)	$2283 ($2192, $2374)	$910 ($893, $926)

Receiving physical therapy first compared to early and delayed led to a lower odds of having a knee radiograph (AOR = 0.09; 95% CI 0.06, 0.14), advanced imaging (AOR = 0.31; 95% CI 0.20, 0.46), opioid prescription (AOR = 0.33; 95% CI 0.18, 0.59), muscle relaxant prescription (AOR = 0.37, 95% CI 0.16, 0.76), NSAIDs prescription (AOR = 0.30; 95% CI 0.16, 0.52), other analgesic prescription (AOR = 0.32; 95% CI 0.13, 0.69), and corticosteroid injection (AOR = 0.26; 95% CI 0.10, 0.55) after adjusting for sex and comorbidities. Individuals receiving early physical therapy compared to delayed physical therapy were at lower odds of having a knee radiograph (AOR = 0.48; 95% CI 0.34, 0.66, advanced imaging (AOR = 0.49; 95% CI 0.37, 0.65), opioid prescription (AOR = 0.53; 95% CI 0.37, 0.75), muscle relaxant prescription (AOR = 0.61; 95% CI 0.39, 0.96), NSAIDs prescription (AOR = 0.58; 95% CI 0.42, 0.81), other analgesics prescription (AOR = 0.52; 95% CI 0.32, 0.83), and corticosteroid injection (AOR = 0.46; 95% CI 0.29, 0.62). Odds of healthcare use were also lower for those who did not receive physical therapy and subgroups receiving exercise therapy or only passive interventions (e.g. modalities) if provided by a physical therapist, both within the first 30 days compared to 31 to 90 days after diagnosis. The odds of muscle relaxant use was higher if the individual received only passive interventions (AOR = 1.02; 95% CI 0.32, 3.52). There was a higher likelihood of additional healthcare use in all categories we assessed if individuals received delayed physical therapy compared to early physical therapy. Table 3 provides all healthcare use OR and AOR.

**Table 3:** Odds ratio for healthcare use based on timing

	Unadjusted odds ratio with 95% CI	Adjusted odds ratio with 95% CI
**Physical therapy first**		
Knee radiograph	0.13 (0.12, 0.15)	0.09 (0.06, 0.14)
Advanced imaging	0.37 (0.33, 0.41)	0.31 (0.20, 0.46)
Opioids	0.35 (0.30, 0.42)	0.33 (0.18, 0.59)
Muscle relaxants	0.37 (0.29, 0.48)	0.37 (0.16, 0.76)
NSAIDs	0.33 (0.28, 0.38)	0.30 (0.16, 0.52)
Other analgesics (acetaminophen, etc.)	0.38 (0.29, 0.49)	0.32 (0.13, 0.69)
Corticosteroid injections	0.37 (0.30, 0.45)	0.26 (0.10, 0.55)
**Early physical therapy**		
Knee radiograph	0.59 (0.55, 0.65)	0.48 (0.34, 0.66)
Advanced imaging	0.53 (0.49, 0.57)	0.49 (0.37, 0.65)
Opioids	0.68 (0.60, 0.76)	0.53 (0.37, 0.75)
Muscle relaxants	0.80 (0.68, 0.94)	0.61 (0.39, 0.96)
NSAIDs	0.73 (0.66, 0.81)	0.58 (0.42, 0.81)
Other analgesics (acetaminophen, etc.)	0.73 (0.61, 0.87)	0.52 (0.32, 0.83)
Corticosteroid injections	0.68 (0.59, 0.78)	0.46 (0.29, 0.62)
**Delayed physical therapy**		
Knee radiograph	1.68 (1.55, 1.83)	2.11 (1.53, 2.94)
Advanced imaging	1.90 (1.75, 2.05)	2.03 (1.54, 2.68)
Opioids	1.48 (1.32, 1.66)	1.90 (1.33, 2.70)
Muscle relaxants	1.25 (1.07, 1.47)	1.64 (1.04, 2.58)
NSAIDs	1.37 (1.24, 1.51)	1.73 (1.23, 2.41)
Other analgesics (acetaminophen, etc.)	1.36 (1.15, 1.62)	1.94 (1.21, 3.10)
Corticosteroid injections	1.48 (1.29, 1.70)	2.20 (1.40, 3.46)
**No physical therapy**		
Knee radiograph	0.61 (0.59, 0.64)	0.52 (0.47, 0.61)
Advanced imaging	0.55 (0.53, 0.47)	0.55 (0.48, 0.63)
Opioids	0.55 (0.52, 0.59)	0.55 (0.45, 0.67)
Muscle relaxants	0.57 (0.52, 0.62)	0.52 (0.41, 0.67)
NSAIDs	0.53 (0.50, 0.55)	0.54 (0.45, 0.65)
Other analgesics (acetaminophen, etc.)	0.54 (0.49, 0.60)	0.53 (0.41, 0.69)
Corticosteroid injections	0.57 (0.53, 0.62)	0.56 (0.43, 0.72)
**Exercise therapy**		
Knee radiograph	0.37 (0.34, 0.40)	0.30 (0.22, 0.40)
Advanced imaging	0.47 (0.44, 0.51)	0.48 (0.37, 0.62)
Opioids	0.59 (0.53, 0.66)	0.55 (0.39, 0.78)
Muscle relaxants	0.65 (0.56, 0.76)	0.52 (0.34, 0.82)
NSAIDs	0.62 (0.56, 0.68)	0.58 (0.42, 0.81)
Other analgesics (acetaminophen, etc.)	0.62 (0.53, 0.74)	0.53 (0.34, 0.84)
Corticosteroid injections	0.58 (0.51, 0.66)	0.40 (0.26, 0.61)
**Passive interventions only**		
Knee radiograph	0.37 (0.29, 0.47)	0.42 (0.17, 1.01)
Advanced imaging	0.46 (0.37, 0.58)	0.82 (0.36, 1.87)
Opioids	0.69 (0.50, 0.96)	0.57 (0.21, 1.55)
Muscle relaxants	0.69 (0.45, 1.08)	1.02 (0.32, 3.52)
NSAIDs	0.67 (0.50, 0.89)	0.69 (0.26, 1.81)
Other analgesics (acetaminophen, etc.)	0.72 (0.44, 1.18)	0.56 (0.15, 2.05)
Corticosteroid injections	0.35 (0.24, 0.51)	0.53 (0.12, 2.15)

Covariates were: sex, cardiometabolic syndrome, chronic pain, insomnia, depression, and substance abuse one year prior to diagnosis

Table 4 outlines recurrence rates between groups, and there were statistically significant differences after adjusting for sex and comorbidities. The odds of having at least one more additional recurrence of knee pain were lower if receiving physical therapy first (AOR = 0.55; 95% CI 0.37, 0.79) compared to early or delayed physical therapy or early physical therapy (AOR = 0.56; 95% CI 0.43, 0.74) compared to delayed physical therapy. Those in the delayed physical therapy group were at higher odds of having a recurrence (AOR = 1.78; 95% CI 1.36, 2.33) compared to early physical therapy. If there was no physical therapy use at all, the odds of recurrence were lower (AOR = 0.75; 95% CI 0.66, 0.86). A sensitivity analysis using 14 days as the threshold instead of 30 resulted in no change in healthcare use and recurrence rates for any of the groups.

**Table 4:** Odds of recurrence in knee pain by group

	Physical therapy first (***n*** = 3591)		Early physical therapy (***n*** = 7924)		Delayed physical therapy (***n*** = 3915)		No physical therapy (***n*** = 58,579)	
	OR (95% CI)	*P* value	OR (95% CI)	*P* value	OR (95% CI)	*P* value	OR (95% CI)	*P* value
Unadjusted	0.49 (0.45, 0.54)	**< 0.001**	0.64 (0.59, 0.79)	**< 0.001**	1.57 (1.45, 1.69)	**< 0.001**	0.80 (0.77, 0.83)	**< 0.001**
Adjusted	0.55 (0.37, 0.79)	**0.002**	0.56 (0.43, 0.74)	**< 0.001**	1.78 (1.36, 2.33)	**< 0.001**	0.75 (0.66, 0.86)	**< 0.001**

Covariates were: sex, cardiometabolic syndrome, chronic pain, insomnia, depression, and substance abuse one year prior to diagnosis

## Discussion

The primary purpose of the study was to assess how the use and timing of physical therapy influenced downstream healthcare use and costs in individuals seeking care for PFP. A secondary objective was to determine if the timing of physical therapy was associated with recurrences of knee pain. For individuals with PFP in this cohort who saw a physical therapist, initial contact with the physical therapist as the first provider (same day as PFP diagnosis) was much less common than receiving a referral. Total physical therapy visits were fewest if physical therapy was the initial contact provider, and highest in the delayed physical therapy group. Patients who had physical therapy first or early were less likely to receive additional medical care including knee radiographs, advanced imaging, medications, and corticosteroid injections. Delayed physical therapy was associated with a greater recurrence. These findings align with what has been reported in individuals with low back pain [8].

Most of the cohort using physical therapy received it early (51.4%), between one to 30 days after the initial PFP diagnosis, consistent with other studies that examined timing of physical therapy for low back pain [23–25]. The odds of undergoing imaging or receiving pharmacological treatment was also lower in the early group compared to delayed physical therapy, comparable to other studies (primarily spinal pain) [8]. Healthcare use, physical therapy visits, total knee-related care (visits and costs), and total healthcare costs over the 2 years following the initial PFP diagnosis were higher in the delayed physical therapy group compared to the first or early physical therapy groups. Although previous literature has been heavily focused on the spine, these findings are complementary because patients with PFP follow similar trajectories of chronicity and recurrence [11, 26]. The findings may assist with decisions about use and referral to physical therapy for PFP.

Individuals receiving physical therapy first had the lowest likelihood of additional future healthcare use. While injury severity data are lacking, and it is possible that those accessing physical therapy as first contact for PFP had less severe symptoms or were less likely to have a concurrent medical condition they wanted to discuss with their primary care provider, it is also possible that symptoms were better managed through a short course of initial physical therapy treatment. In a traditional health system, receiving physical therapy on the same day as diagnosis through first contact with a physical therapist is not as common, so these results may not be typical outside of the MHS. However, these results may help facilitate a push toward more efficient pathways for appropriate care in individuals with PFP. Additionally, 30.0% (*n* = 1078) of individuals who had first contact with a physical therapist had only one subsequent knee-related care visit in the following 2 years, compared to 22.5% (*n* = 683) who were seen by a different healthcare provider type. This suggests that physical therapists were effective with their interventions, and were not likely to provide potentially unnecessary treatment [6].

It is important to note that less than 25% of the cohort utilized any physical therapy, and those who did not receive physical therapy had low recurrence rates, and low total healthcare and knee-related costs. This could reflect patients with less severe symptoms, those with higher self-efficacy that preferred self-management strategies, or those for whom making additional physical therapy visits was not feasible (e.g., military training, deployments). Physical therapy, like most other interventions, may not be the best or necessary intervention for every single patient [27]. For patients where physical therapy appears to have merit as part of the treatment plan, either by the patient or the diagnosing clinician, the timing of physical therapy appears to be highly relevant.

A higher percentage of individuals receiving delayed physical therapy were ordered knee radiographs, compared to those with earlier physical therapy, suggesting that the ordering of a radiograph may have delayed decisions regarding physical therapy referrals. Radiographs are discouraged for non-traumatic knee pain, as current research demonstrates they often do not correlate with symptoms, are a potential waste of resources and can increase healthcare costs, and knowledge of results often lead to a poorer prognosis [28–30]. Delayed physical therapy is typical in a traditional healthcare system whereby individuals do not see a physical therapist or other healthcare provider until later in their care pathway [6], so the use of additional testing prior to receiving physical therapy, including radiographs, may not be surprising yet does not match current recommendations discouraging the use of radiographs. In fact, radiographs were the most commonly used healthcare use outcome in all groups in our cohort. This finding merits further investigation in future study of healthcare practices around the management of PFP. The use of early radiographs was common practice for patients in this cohort, influenced by the use and/or timing of physical therapy, and in conflict with current recommendations [29–31].

For patients who received physical therapy, the AOR of having at least one recurrence in the 2 year surveillance period was 78% higher in the delayed physical therapy group compared to the early physical therapy group. Conversely, receiving physical therapy first or early led to a lower odds of having a recurrence. This speaks to the importance of receiving appropriate interventions, including physical therapy. A quicker reduction of symptoms in patients with PFP leads to better outcomes in individuals in which the initial duration is shorter before receiving care [11, 26]. Although we do not know the duration of the symptoms before individuals sought care, they could not have received any knee-related care in the year prior to diagnosis to be included in the cohort.

Alternatively, individuals receiving physical therapy first or early may have received home exercise programs (HEPs) along with their supervised visits and ultimately were able to manage their symptoms at home with a HEP after only a small number of supervised visits. There is evidence suggesting adherence to HEPs is better than supervised visits in patients with PFP [32], so perhaps this cohort opted for a HEP. Lastly, it is possible these individuals’ symptoms resolved entirely.

Similar to the timing of physical therapy, early exercise therapy from a physical therapist led to lower odds of healthcare use. Exercise therapy is widely recommended in recent guidelines [4, 5], but there is limited evidence for PFP on specific dosing variables that improve outcomes [33], or how the timing of exercise impacts outcomes. Although there are no specifics on the exact exercises that were incorporated, this finding is promising because it supports the idea that early appropriate interventions can decrease the need for follow-up care, including imaging or medications. Also, the odds for downstream healthcare use were lower in those who received only early passive interventions from a physical therapist. Passive interventions are not recommended for PFP [5], especially in isolation, but this finding implies that if passive interventions were used, the timing of these isolated passive interventions is important for individuals with PFP.

### Limitations

This study has several limitations. First, observational studies limit inferences about causation. Other confounding variables beyond our ability to control may provide greater explanation for differences in outcomes related to the timing of physical therapy. Second, the MHS is a single-payer government system and results may differ in other settings. Third, a common limitation with studies of this nature are assumptions made about coding accuracy in medical records. It is possible there was variability in coding, which we tried to control for as much as possible in our methods, but there is the potential that some cases of PFP were not included in the cohort and some cases that were not true PFP were included. Codes for knee-related care and knee pain included other knee diagnoses, primarily to capture variations in diagnostic labels used across the health system (e.g. the generic “pain in joint, lower leg” which is often used for anterior knee pain). This increases the sensitivity for including relevant care events, but comes at a cost of specificity. However, we did try to control for this as much as possible by removing individuals from this cohort who had knee surgeries or a different follow-on diagnosis within 6 months (cruciate or meniscus injury, fracture, osteoarthritis, patellar tendinopathy, iliotibial band friction syndrome) in case the original diagnostic label was incorrect. This cohort was also limited to individuals with a full 2 years of follow-up available, and therefore patterns of care when including all patients with PFP regardless of follow-up availability could be different. Each of these limitations could impact the overall size and specificity of the cohort. Fourth, it is possible care was sought outside the MHS, which is not captured in this database. However, most beneficiaries (all active duty) would have no co-pay required in this system and therefore seeking outside care is unlikely. While large healthcare databases often have missing data for this reason [34], these data are from a closed single-payer health system, with an internal 90-day validation period which allows for very little missing data. Fifth, practice patterns may have changed since the collection of this data. Lastly, patient-reported outcomes would have provided the ability to better infer levels of pain, disability, and function, which could significantly influence the outcomes.

## Conclusions

For patients receiving physical therapy for PFP, timing is important. Approximately 75% received it within 30 days of their initial PFP diagnosis. Healthcare use, costs and odds of recurrence were lower for patients who had physical therapy as their first contact (day zero) or within 30 days compared to having physical therapy between 31 and 90 days after diagnosis. These results are similar to those found in other musculoskeletal conditions and highlight the need for further prospective studies assessing timing of physical therapy.

## Data Availability

The datasets generated and/or analysed during the current study are not publicly available due to being proprietary to the US Defense Health Agency but are available from the corresponding author on reasonable request.
